# MicroRNA Expression Profile Changes in the Leukocytes of Parkinson’s Disease Patients

**DOI:** 10.32607/actanaturae.11729

**Published:** 2022

**Authors:** N. S. Ardashirova, N. Yu. Abramycheva, E. Yu. Fedotova, S. N. Illarioshkin

**Affiliations:** Research Center of Neurology, Moscow, 125367 Russia

**Keywords:** Parkinson’s disease, microRNA, biomarkers

## Abstract

Parkinson’s disease (PD) is one of the most common movement disorders. It is primarily diagnosed clinically. A correct diagnosis of PD in its early stages is important for the development of a pathogenic treatment, which necessitates a search for potential biomarkers of the disease. We evaluated the diagnostic value of several microRNAs and their relationship with the clinical characteristics of PD. The study included 70 PD patients and 40 healthy volunteers. We analyzed the expression of 15 microRNAs in blood leukocytes, which were selected based on literature data and modern concepts of molecular PD pathogenesis. All patients were evaluated using the Hoehn and Yahr scale, UPDRS, NMSQ, and PDQ-39. The data analysis revealed a statistically significant increase in the expression of miR-7-5p, miR-29c-3p, and miR-185-5p and a statistically significant decrease in the expression of miR-29a-3p and miR-30c-1-5p in leukocytes in PD. However, the altered microRNA profile was shown to have a moderate diagnostic value for PD diagnosis. MicroRNA expression changes were associated with the motor and non-motor phenotypic features of PD and administration of anti-Parkinson’s drugs. Also, a relationship between some of the microRNAs studied and the duration and severity of PD was found, which may potentially be used to monitor disease progression.

## INTRODUCTION

Parkinson’s disease (PD) is one of the most common movement disorders and a serious medical and social problem. According to modern concepts, PD belongs to the synucleinopathies, a group of disorders characterized by the formation of pathological alpha-synuclein aggregates in the central and peripheral nervous system [1], also including dementia with Lewy bodies, multiple system atrophy, and isolated autonomic failure. An important predictor of synucleinopathies is a REM sleep behavior disorder [1].

Currently, PD is primarily diagnosed clinically. In this case, even movement disorder experts are able to make a correct clinical diagnosis of PD using pathologic findings in just approximately 80% of the cases [2]. Contemporary methods of radionuclide neuroimaging (positron emission and single photon emission-computed tomography) can be used to accurately assess dopaminergic pathways and, thereby, significantly improve the accuracy of the PD diagnosis [3]. However, these are expensive procedures that are associated with radiation exposure and they cannot be used to differentiate PD from atypical parkinsonian syndromes [4]. The insufficient accuracy that characterizes a life-time diagnosis, especially in the early stages of PD, is considered one of the important causes behind the failure of the drug trials utilized in the pathogenic treatment of PD [5]. Therefore, the development of informative and accessible diagnostic biomarkers of PD is critical.

The molecular pathogenesis of PD is complex. One of its components is presumably an impaired epigenetic regulation of gene expression, which involves microRNAs [6]. To date, more than 5,000 different microRNAs have been identified in the human genome (http://www.mirbase.org). The studied mechanism of microRNA action involves the implementation of RNA silencing. In the RNA-induced silencing complex (RISC), microRNA binds to the 3’-end of a complementary mRNA, leading to mRNA degradation and the prevention of protein translation [7]. There are other mechanisms of expression regulation which involve microRNAs [8]. Importantly, a single microRNA can bind to more than 200 different mRNAs, thus inducing shifts in the regulation of protein cascades [9]. These changes may be the basis for various pathological processes, including those leading to neurodegeneration.

In PD, shifts in blood microRNA levels may reflect, for the most part, the involvement of numerous organs and systems in the pathological process. It is the multiple organs pathology that is believed to cause the development of the non-motor (gastrointestinal, cardiac, etc.) manifestations that are so characteristic of PD. Therefore, better understanding of the features of blood microRNA levels could help us develop a new informative PD biomarker for an early and differential diagnosis of the disease, prediction of its course, a more accurate assessment of the motor and non-motor manifestation ratio, etc.

The possibility of using certain microRNAs in the diagnosis of PD has already been considered [10 , 11, 12]. Some studies have tested panels of several microRNAs as a PD biomarker [13 , 14, 15, 16]. However, it should be taken into account that the microRNA profile is quite dynamic and is affected by various factors. For example, the microRNA profile has been shown to be influenced by ongoing anti-Parkinson’s therapy [17 , 18, 19, 20, 20] and deep brain stimulation [21]. Based on the analysis of published data, we selected 15 microRNAs whose expression in the blood and brain of PD patients differed significantly from that of the controls in at least two studies.

In this study, we evaluated the significance of the selected microRNAs for a PD diagnosis and their correlation with the clinical characteristics of this disease.

## EXPERIMENTAL

The study included 70 PD patients and 40 healthy volunteers. The PD group comprised 35 males and 35 females (mean age, 60.5 ± 11.8 years). Study and control group patients were comparable in gender and age. The study was approved by the local ethical committee of the Research Center of Neurology. All participants signed an informed consent.

The diagnosis of PD was made according to the International Parkinson and Movement Disorder Society (MDS) criteria [22]. The age of onset was 53 ± 13 years, with a disease duration of 6.4 ± 7.0 years. The mixed form of PD was diagnosed in 52 (74.3%) patients, and the akinetic-rigid form of PD was present in 18 (25.7%) patients. The mean score of clinical symptom severity on the Unified Parkinson’s Disease Rating Scale (UPDRS) was 65.6 ± 27.1, and the mean Hoehn and Yahr stage of PD was 2.4 ± 0.9.

All patients completed the Non-Motor Symptoms Questionnaire (NMSQ), with the mean score of 9.0 ± 5.2. The patients underwent testing using the Hospital Anxiety and Depression Scale (HADS) (mean scores of 6.4 ± 4.0 and 7.0 ± 4.6 for anxiety and depression, respectively) and the Montreal Cognitive Assessment scale (MoCA) (mean score of 23.1 ± 4.2). The patients assessed their quality of life using the Parkinson’s Disease Questionnaire (PDQ-39) (mean score of 44 ± 30).

Most of the PD patients received anti-Parkinson’s drugs, including levodopa (41 patients, 58.6%), dopamine receptor agonists (30 patients, 42.6%), and amantadine drugs (20 patients, 28.6%). Twenty-three patients (32.9%) did not receive any therapy at the time of enrollment.

We studied the following 15 microRNAs: miR-7-1-5p, miR-24-1-3p, miR-29a-3p, miR-29c-3p, miR-30c-1-5p, miR-106a-5p, miR -126-3p, miR-129-1-5p, miR-132-3p, miR-135b-5p, miR-146a-5p, miR-185-5p, miR-214-3p, miR-221-3p, and miR -520d-5p.

The leukocyte fraction was isolated from the venous blood of all subjects. Then, total RNA was isolated using a RNeasy mini kit (Qiagen) according to the manufacturer’s standard protocol. After RNA isolation, reverse transcription specific to each microRNA was performed using stem-loop primers and a reverse transcription kit (Syntol). The relative concentration of each RNA was determined during a real-time polymerase chain reaction using the appropriate kit (Syntol); miR-191-5p was used as a reference RNA. The RNA concentration was calculated using the 2(–ΔΔC(T)) method.

Statistical processing was performed using the SPSS and Statistica 10.0 software. The Shapiro– Wilk test was used to check if the variable followed a normal distribution. Due to the distribution of microRNA values not being normal, the nonparametric Mann–Whitney, Kruskal–Wallis, and Spearman correlation coefficient tests were used. We also used a logistic regression analysis and ROC analysis. The statistical significance level was set to 0.05.

## RESULTS

level of three microRNAs – miR-7-1-5p, miR-29c-3p, and miR-185-5p – and a statistically significant decrease in the expression level of two microRNAs – miR-29a-3p and miR-30c-1-5p – were revealed in the PD group compared to the control group (Table). However, despite the statistical significance of the differences detected, there was a significant overlap in the relative expression levels in these groups.

**Table:** MicroRNA expression in PD patients and healthy volunteers

microRNA	Parkinson’s disease	Control group	p (U)
miR-7-1-5p	0.68 [0.19; 1.7]	0.2 [0.04; 1.5]	0.024*
miR-24-1-3p	455.72 [0.43; 654.6]	480.88 [0.83; 602.4]	0.684
miR-29a-3p	0.63 [0.41; 1.01]	0.97 [0.66; 1.4]	0.003**
miR-29c-3p	1.76 [0.93; 3.58]	0.77 [0.59; 1.98]	0.003**
miR-30c-1-5p	0.53 [0.34; 1.43]	1.03 [0.46; 1.77]	0.043*
miR-106a-5p	1.41 [0.43; 3.5]	1.39 [0.76; 2.8]	0.691
miR-126-3p	0.23 [0.15; 0.44]	0.4 [0.11; 0.8]	0.194
miR-129-1-5p	0.47 [0.2; 2.21]	0.4 [0.23; 0.71]	0.403
miR-132-3p	1.01 [0.4; 2.01]	0.87 [0.37; 1.39]	0.209
miR-135b-5p	54.5 [4.02; 2479.78]	284.29 [1.02; 149791.83]	0.946
miR-146a-5p	0.11 [0.03; 1.37]	0.07 [0.03; 0.34]	0.337
miR-185-5p	13631.02 [380.56; 21875.07]	863.02 [0.17; 14684.43]	0.017*
miR-214-3p	15.23 [6.97; 22.65]	15.75 [6.01; 27.3]	0.709
miR-221-3p	0.63 [0.42; 1.04]	0.72 [0.49; 0.99]	0.443
miR-520d-5p	0.27 [0.05; 1.02]	0.52 [0.04; 1.77]	0.374

^*^p < 0.05;

^**^**p < 0.01.

All cases where p < 0.05 are shown in bold.

**Fig. 1:**
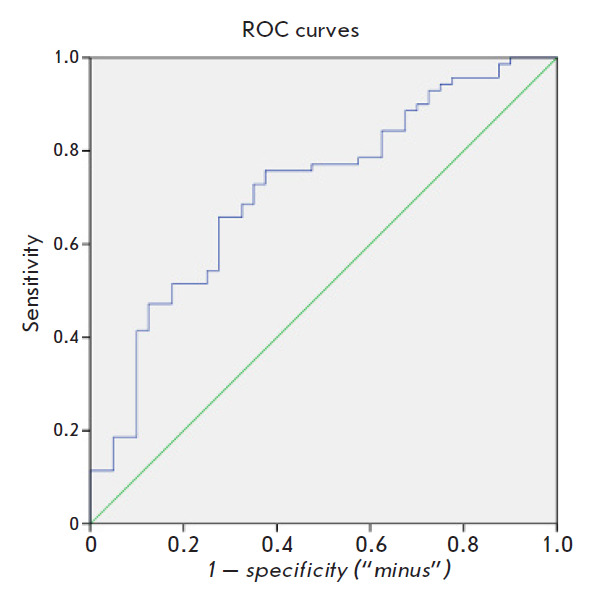
ROC analysis of a logistic regression model with miR-29c-3p and miR-185-5p for the diagnosis of Parkinson’s disease

Next, we assessed the possibility of using individual microRNAs as PD biomarkers. The ROC analysis revealed that some microRNAs could be used to differentiate PD from the controls: miR-7-1-5p (AUC = 0.63, p = 0.024; 95% CI 0.517–0.742), miR-185-5p (AUC = 0.638; p = 0.016; 95% CI 0.53–0.744), miR-29c-3p (AUC = 0.673; p = 0.003; 95% CI 0.56–0.778). However, the sensitivity and specificity of these biomarkers are obviously insufficient for a PD diagnosis. A logistic regression analysis using the backward Wald method was employed to search for the most optimal microRNA combination that could possess the highest informative value as a biomarker. The combination of miR-29c-3p and miR-185-5p proved to be the most informative. During the subsequent ROC analysis, the area under the curve was 0.715 (Fig. 1). Thus, this two-microRNA model allows a 71.5% probability of differentiating PD patients from healthy individuals.

We studied the relationship between microRNA expression and the clinical characteristics of PD. No significant correlations between the microRNA levels and the age of onset were found, besides one weak correlation (R < |0.3|) between the age at study entry and miR-135b-5p. The analysis of any correlations between microRNA levels and disease duration revealed six weak but significant correlations (miR-132-3p, miR-146a-5p, miR-106a-5p, miR-24-1-3p, miR-29a-3p, miR-30c -1-5p) and two moderate correlations (miR-126-3p, R = 0.316; p = 0.07 and miR-129-1-5p, R = 0.385; p = 0.001). These microRNAs may serve as markers of disease progression.

The analysis of the microRNA expression in different PD forms showed that the miR-29a-3p level in the akinetic-rigid form was significantly higher than that in the mixed form, 1.06 [0.6; 1.59] and 0.6 [0.43; 0.85] (p = 0.018), respectively. A negative correlation was found between the miR-30c-1-5p level and the Hoehn and Yahr disease stage (R = –0.303; p = 0.19). At the same time, a differential expression of these two microRNAs (Table) was detected in PD and the control groups. No correlation was found between microRNA levels and the total UPDRS (including subscales) score. A negative correlation was revealed between the miR-106a-5p level and the total NMSQ score (R = –0.358, p = 0.011). No correlations were found with HADS scores. Also, there were no correlations with the cognitive impairment severity (MoCA scale).

The miR-29a-3p expression (p = 0.045) was statistically significantly reduced in patients treated with levodopa. The patients receiving both dopamine receptor agonists and amantadine demonstrated a decrease in miR-7-1-5p in (p = 0.0048 and p = 0.037, respectively). Interestingly, the expression of both miR-7-1-5p and miR-29a-3p was different in PD patients and the controls (Table).

## DISCUSSION

To date, a significant number of studies on the biomarker potential of microRNAs in PD have been performed. However, the results remain mostly contradictory, due to the variety of the blood components (plasma, leukocytes, serum, vesicles) being studied and the wide range of microRNAs analyzed, not to mention the different methods used to detect them [23].

In this study, we used 15 microRNAs that had been shown in previous studies to exhibit significant expression differences between PD and the controls. According to our data, the combination of two microRNAs – miR-29c-3p and miR-185-5p – has the greatest diagnostic value in PD. A significant increase in the miR-7-1-5p level and a significant decrease in miR-29a-3p in PD were also observed, with these levels being significantly affected by antiparkinsonian therapy, as shown in our work. In addition, a significant decrease in miR-30c-1 in PD was revealed. The level of this microRNA steadily decreased as the disease progressed, and its severity increased according to the Hoehn–Yahr functional scale, suggesting that it can be used as a marker of disease progression (i.e., a marker of advanced PD stages).

However, a number of other microRNAs, with levels similar to those of the controls, appeared to be associated with non-motor manifestations of PD (miR-106a-5p) and disease duration (miR-126-3p, miR-129-1-5p). Thus, while not being direct biomarkers of the disease, these microRNAs may be valuable in more accurately identifying non-motor phenotypes of PD and more objectively monitoring the course of the disease.

The role of miR-185 in PD was analyzed in several studies. A decrease in the miR-185 level was observed in MPTP-treated SH-SY5Y dopaminergic neuroblastoma cells, while increased miR-185 expression reduced MPTP-induced apoptosis and autophagy [24]. A study by Rahimmi et al. in SH-SY5Y cells and Wistar rats with rotenone-induced parkinsonism revealed that inhibition of miR-185 expression with a specific small interfering RNA leads to a significant increase in the LRRK2 gene expression. This gene plays an important role in the pathogenesis of PD, with its mutations leading to the development of hereditary forms of PD [25]. A decrease in miR-185 and an increased LRRK2 expression were demonstrated in the substantia nigra and striatum of animal models. A study by Briggs et al. also reported miR-185 expression changes in the substantia nigra, but in the opposite direction [26]. The use of miR-185 as a biomarker was evaluated in several studies, and two of them revealed a decreased expression level of this microRNA in PD, compared with that in the control group [15, 27]. In contrast, our study has shown an increased expression of this potential marker. Thus, the data on the miR-185 expression level in PD remain inconclusive.

The miR-29 family includes three microRNAs: miR-29a, miR-29b, and miR-29c. The studies on the use of these microRNAs as biomarkers have repeatedly demonstrated decreased miR-29a and miR-29c expressions in the blood of PD patients, with the miR-29a and miR-29c expressions tending to decrease with the disease severity (Hoehn and Yahr scale) [28]. A prospective study of patients at risk of synucleinopathies revealed decreased miR-29a and miR-29c levels in patients with REM sleep behavior disorders who were subsequently diagnosed with synucleinopathy [29]. Several studies investigated miR-29a alone and demonstrated a decrease in its level, which is consistent with our results [14, 30, 31]. Serafin et al. reported an increased miR-29a expression only in levodopa-treated patients and did not find changes in untreated patients [19]. Our study has also revealed decreased miR-29a levels upon levodopa therapy.

The increased miR-29c expression found in PD patients in the Turkish population [32] is consistent with our results, but it contradicts most of the available data [12, 14, 31]. The targets of this highly pathogenetically promising family of microRNAs include mRNAs of the PARK-7 (DJ-1) gene, whose mutations can lead to PD, GPR37 mRNA, with its substrate being the parkin protein associated with the development of early PD, and various regulators of apoptosis processes (Puma, Bim, Bak, Bcl2, IGF1, AKT1). The targets of individual microRNAs from the miR-29 family can significantly overlap, but their role in the pathogenesis of various PD forms is beyond doubt.

MiR-7 was reported to reduce the expression of alpha-synuclein [33, 34], the impaired processing of which is considered one of the key components of PD pathogenesis. One study demonstrated a decreased miR-7 expression in the brain of PD patients, probably resulting in the increased expression of alpha-synuclein [6]. In addition, a decreased miR-7 expression was shown to increase the risk of apoptosis and inhibit the growth of dopaminergic neurons in culture [35]. In our study, on the contrary, the expression level of miR-7 in the PD group was significantly higher than that in the control group. Alieva et al. also indicated a many-fold increase in the miR-7 expression in a subgroup of PD patients receiving anti-Parkinson’s drugs [18]. Our study did not find any effect of the levodopa therapy on the miR-7 level, while administration of dopamine receptor agonists and amantadines was associated with a decreased miR-7 expression. Conflicting results on the miR-7 level in PD and the effect of anti-Parkinson’s therapy on it require further clarification.

The decrease in the miR-30c-1 level in PD reported in the studies by Vallelunga et al. and Martins et al. is consistent with our results [31, 36]. No direct effect of miR-30c-1 on the expression of the genes responsible for the development of PD was found. However, according to various databases, the putative targets of this microRNA (Notch1, HDAC4, BECN1, UBE2I, HSPA4, and DNMT1) were shown to play a role in regulating the autophagy and apoptosis of dopaminergic cells [37].

## CONCLUSION

To summarize, we have shown that the combination of two microRNAs (miR-29c-3p and miR-185-5p) can be considered a potential PD biomarker, with moderate diagnostic significance. The expression level of several microRNAs has been found to reflect the clinical characteristics of PD, to depend on the disease duration and stage and ongoing therapy, serve as a marker of the disease form, and be associated with the severity of non-motor manifestations and the quality of life of PD patients. According to published data, some of the diagnostic microRNAs are associated with certain components of PD pathogenesis. Our results are preliminary and require further research.

## Abbreviations

PD: Parkinson’s disease
REM-sleep: rapid eye movement sleep
RNA: ribonucleic acid
UPDRS: unified Parkinson’s disease rating scale
NMSQ: non-motor symptoms questionnaire
HADS: hospital anxiety and depression scale
MoCA: Montreal cognitive assessment
PDQ-39: Parkinson’s disease questionnaire

## References

[1] Coon E.A., Singer W. (2020). CONTINUUM: Lifelong Learning in Neurology..

[2] Rizzo G., Copetti M., Arcuti S., Martino D., Fontana A., Logroscino G. (2016). Neurology..

[3] Gerasimou G., Costa D.C., Papanastasiou E., Bostanjiopoulou S., Arnaoutoglou M., Moralidis E., Aggelopoulou T., Gotzamani-Psarrakou A. (2012). Ann. Nucl. Med. Japan..

[4] Arena J.E., Stoessl A.J. (2016). Parkinsonism and Related Disorders..

[5] Lang A.E., Espay A.J. (2018). Movement Disorders..

[6] Tatura R., Kraus T., Giese A., Arzberger T., Bucholz M., Höglinger G., Muller U. (2016). Parkinsonism and Related Disorders..

[7] Wahid F., Shehzad A., Khan T., Kim Y.Y. (2010). Biochim. Biophys. Acta – Mol. Cell Res..

[8] Mathonnet G., Fabian M.R., Svitkin Y.V., Parsyan A., Huck L., Murata T., Biffo S., Merrick W., Darzynkiewicz E., Pillai R.S. (2007). Science..

[9] Leggio L., Vivareli S., L’Episcopo F., Tirolo C., Caniglia S., Testa N., Barchetti B., Iraci N. (2017). Int. J. Mol. Sci..

[10] Ma F., Zhang X., Yin K.J. (2020). Exp. Neurol..

[11] Cao X., Lu J.M., Zhao Z.Q., Li M.C., Lu T., An X.S., Xue L.J. (2017). Neurosci. Lett..

[12] Ma W., Li Y., Wang C., Xu F., Wang M., Liu Y. (2016). Cell. Biochem. Funct..

[13] Kean S., Petillo D., Kang U.J., Resau J.H., Berryhill B., Linder J., Forsgren L., Neuman L.A., Tan A.C. (2012). J. Parkinson’s Dis..

[14] Botta-orfila T., Morato X., Compta Y., Lozano J.J., Falgas N., Valldeoriola F., Pont-Sunyer C., Vilas D., Mengual L., Fernandez M. (2014). J. Neurosci. Res..

[15] Ding H., Huang Z., Chen M., Wang C., Chen X., Chen J., Zhang J. (2016). Parkinsonism Related Disorders J..

[16] Dong H., Wang C., Lu S., Yu C., Huang L., Feng W., Xu H., Chen X., Zen K., Yan Q. (2016). Biomarkers..

[17] Margis R.R., Margis R.R., Rieder C.R.M. (2011). J. Biotechnol..

[18] Alieva A., Filatova E.V., Karabanov A.V., Illarioshkin S.N., Limborska S.A., Shadrina M.I., Slominsky P.A. (2014). Parkinsonism Related Disorders J..

[19] Serafin A.., Foco L., Zanigni S., Blankenburg H., Picard A., Zanon A., Gianni G., Pichler I., Maurizio F.F., Cortell P., Pramstaller P.P., Hicks A.A., Domingues F.S., Schwienbacher C. (2015). Neurology..

[20] Caggiu E., Paulus K., Mameli G., Arru G., Sechi G. P., Sechi L.A. (2018). eNeurologicalSci..

[21] Soreq L., Salomonis N., Bronstein M., Greenberg D.S., Israel Z., Bergman H., Soreg H. (2013). Front. Mol. Neurosci..

[22] Postuma R., Berg D., Stern M., Poewe W., Olanow C.W., Oertel W., Obeso J., Marek K., Litvan I., Lang A. (2015). Mov. Disord: Official J. Mov. Disord. Soc..

[23] Nies Y.H., Mohamad Najib N.H., Lim W.L., Kamaruzzaman M.A., Yahaya M.F., Teoh S.L. (2021). Front. Neurosci..

[24] Wen Z., Zhang J., Tang P., Tu N., Wang K., Wu G. (2018). Mol. Med. Rep..

[25] Rahimmi A., Peluso I., Rajabi A., Hassanzadeh K. (2019). Oxid. Med. Cell. Longev. Hindawi..

[26] Briggs C.E., Wang Y., Kong B., Woo T.U., Iyer L.K., Sonntag K.C. (2015). Brain Res..

[27] Chen L., Yu Z. (2018). Brain Behav..

[28] Bai X., Tang Y., Yu M., Wu L., Liu F., Ni J., Wang Z., Wang J., Fei J., Wang W. (2017). Sci. Rep..

[29] Fernández-Santiago R., Iranzo A., Gaig C., Serradell M., Fernández M., Tolosa E., Santamaría J., Ezquerra M. (2015). Ann Neurol..

[30] Barbagallo C., Mostile G., Baglieri G., Giunta F., Luca A., Raciti L., Zappia M., Purrello M., Ragusa M., Nicoletti A. (2020). Cell. Mol. Neurobiol..

[31] Martins M., Rosa A., Guedes L.C., Fonseca B.V., Gotovac K., Violante S., Mestre T., Coelho M., Rosa M.M., Martin E.R. (2011). PLoS One..

[32] Ozdilek B., Demircan B. (2020). Int. J. Neurosci..

[33] Doxakis E. (2010). J. Biol. Chem..

[34] Junn E., Lee K.W., Jeong B.S., Chan T.W., Im J.Y., Mouradian M.M. (2009). Proc. Natl. Acad. Sci. USA..

[35] Li S. Lv X., Zhai K., Xu R., Zhang Y., Zhao S., Qin X., Yin L., Lou J. (2016). Am. J. Transl. Res..

[36] Vallelunga A., Ragusa M., Di Mauro S., Iannitti T., Pilleri M., Biundo R., Weis L., Di Pietro C., De Iuliis A., Nicoletti A. (2014). Front. Cell. Neurosci..

[37] Vallelunga A., Iannitti T., Dati G., Capece S., Maugeri M., Tocci E., Picillo M., Volpe G., Cozzolino A., Squillante M. (2019). Mol. Biol. Rept..

